# Dynamic Changes in Microbial Communities, Physicochemical Properties, and Flavor of Kombucha Made from Fu-Brick Tea

**DOI:** 10.3390/foods12234242

**Published:** 2023-11-24

**Authors:** Xiaoya Wu, Yue Zhang, Baoshan Zhang, Honglei Tian, Yan Liang, Hui Dang, Yu Zhao

**Affiliations:** 1 College of Food Engineering and Nutritional Science, Shaanxi Normal University, Xi’an 710119, China; a18706819613@163.com (X.W.); zhangyue02172022@163.com (Y.Z.); hongleitian@snnu.edu.cn (H.T.); dangh1202@163.com (H.D.); 2 Research Center of Fruit and Vegetable Deep-Processing Technology, Xi’an 710119, China; baoshan2@snnu.edu.cn; 3 Xianyang Jingwei Fu Tea Co., Ltd., Xianyang 712044, China; lianyan5517@126.com; 4 Key Laboratory of Fu Tea Processing and Utilization, Ministry of Agriculture and Rural Affairs, Xianyang 712044, China

**Keywords:** kombucha, Fu-brick tea, microbial communities, volatile components

## Abstract

In this study, Fu-brick tea (FBT) was used for kombucha preparation. The succession of microbial community structures, changes in physicochemical properties, and the volatiles were investigated during the kombucha fermentation. The sequencing analysis showed that *Komagataeibacter* was the most predominant bacterium. *Aspergillus* and *Zygosaccharomyces* were the dominant fungi before fermentation whereas *Zygosaccharomyces* and *Derkella* were the dominant fungi after 3 days of fermentation. The physicochemical analysis revealed that acetic acid, glucuronic acid, and polyphenols increased by 10.22 g/L, 0.08 g/L, and 177.40 mg/L, respectively, by the end of fermentation. The GC-MS analysis showed that a total of 49 volatile compounds were detected during the fermentation. Moreover, there were great differences in volatile components among the kombucha samples with different fermentation times. Furthermore, the relevance among microbial community and volatile compounds was evaluated through correlation network analysis. The results suggested that *Komagataeibacter*, *Aspergillus*, *Zygosaccharomyces*, and *Dekkera* were closely related to the main volatile compounds of FBT kombucha. The results in this study may provide deep understanding for constructing the microbiota and improving the quality of FBT kombucha.

## 1. Introduction

Kombucha is a traditional fermented beverage. It is produced through the microbial fermentation of sweetened tea using a symbiotic culture of bacteria and yeast (SCOBY) [1,2]. Kombucha has a specific sweet and sour taste. Moreover, it is enriched with a great number of bioactive substances, such as polyphenols, flavones, organic acid, and amino acids, and shows potential health benefits, including liver detoxification, antiobesity, antidiabetic, antimutagenic, and antioxidant effects [3,4,5]. Therefore, kombucha has become popular in recent years.

Most modern studies on kombucha have been conducted to identify its microorganisms and analyze its chemical compounds [6]. Kombucha SCOBY is a complex ecosystem, which primarily consists of yeast, acetic acid bacteria, and lactic acid bacteria. During fermentation, the microbial community varies over time. For instance, *Candida* sp. dominated in the initial kombucha soup than shifted to *Lachancea* sp. on the 7th day of fermentation [7]. Meanwhile, the biochemical properties (like pH, total sugar, total polyphenol, and flavonoids) and flavor constituents also changed during fermentation [8]. Thus, we hypothesize that the shifts in microbial communities have a great role in the change in biochemical compounds and flavor compounds.

Generally, kombucha is prepared using black tea or green tea. Since the demand for kombucha is increasing, some studies have also used other tea leaves, fruit juices, herb infusions, and even the by-products of food industry as alternative substrates for fermenting novel kombucha [4,9,10] In terms of tea, various types of tea such as oolong tea, pu-erh tea, and zijuan tea have been studied in kombucha fermentation [10,11,12]. Fu-brick tea (FBT) is a special dark tea which is mainly produced in China. It stands out due to its creation through the solid-state fermentation of tea leaves with the probiotic *Aspergillus cristatus*, resulting in a distinctive “fungal flower” aroma and a smooth, mellow flavor. Meanwhile, FBT also has health properties, including antiobesity, antioxidant, and antitumor effects [13,14,15].

To the best of our knowledge, there are currently no studies that have used FBT as a substrate to ferment kombucha. Using FBT for kombucha fermentation will introduce the new species *Aspergillus* in kombucha, which may lead to an inevitable impact on the pre-existing microbial community, the biochemical substance, and the flavor in the new kombucha. Therefore, in this study, the never tested substrate FBT was used for kombucha fermentation. The microbial community dynamics and the physical and chemical properties, especially volatile substances, were explored during fermentation. Furthermore, the relationships between core functional microbiota and flavor factors were investigated to improve the quality and control of the new kombucha beverage.

## 2. Materials and Methods

### 2.1. Sample Preparation FBT

Kombucha starter culture was purchased from Hongchajun company (Xi’an, China) and kept at 4 °C in the laboratory. FBT was purchased from Jingwei Fucha Company (Xianyang, China). Kombucha fermentation medium was prepared by adding 15 g of FBT and 300 g of sucrose into 3 L of boiled water, and then it was infused for 10 min. After that, the tea soup was filtered through a sterilized gauze to remove the tea leaves, and distributed equally into 3 sterilized beakers.

The cooled tea soup was inoculated with 10% (*v*/*v*) actively growing tea fungus. The beakers were carefully covered with 8 layers of sterilized gauze and fastened promptly. The fermentation process was conducted at 30 °C for 14 days. Kombucha was taken from the beakers at different fermentation times for 0, 3, 7, 10, and 14 days.

### 2.2. Next Generation Sequencing and Data Analysis

Genomic DNA was extracted from the kombucha samples according to the manufacturer’s instructions (E.Z.N.A.^®^ soil kit, Omega Bio-Tek, Norcross, GA, USA). The concentration and purity of DNA were determined by a spectrophotometer (NanoDrop2000, Repligen, Waltham, MA, USA), and the quality was subsequently checked by 1% agarose gel electrophoresis. The amplified PCR products were recovered using 2% agarose gel electrophoresis. Purification and quantification were performed using an AxyPrep DNA Gel Extraction Kit (Axygen Biosciences, Union City, CA, USA) and QuantiFluor™-ST (Promega, Madison, WA, USA), respectively.

16S rRNA and ITS gene sequencing were used to identify the bacteria and fungus in the kombucha samples, and were performed using an Illumina Miseq PE300 platform. The PE reads obtained from Miseq sequencing were spliced based on the overlap relationships (FLASH 1.2.11 https://ccb.jhu.edu/software/FLASH/index.shtml, accessed on 23 April 2023), and they were quality controlled and filtered using Fastp (version 0.19.6, https://github.com/OpenGene/fastp, accessed on 23 April 2023). The chimeras were removed using USEARCH (version 11, http://www.drive5.com/usearch/, accessed on 25 April 2023). The quality-filtered reads were clustered into OTU with a 97% similarity threshold by UPARSE (version 11, http://drive5.com/uparse/, accessed on 25 April 2023). Representative sequences with the highest abundance were chosen for each OTU and assigned to the bacterial Silva database (Release138 http://www.arb-silva.de, accessed on 25 April 2023) as well as fungus Unite (Release 8.0, http://unite.ut.ee/index.php, accessed on 25 April 2023) using the Ribosomal Database Project classifier (version 2.11, http://sourceforge.net/projects/rdp-classifier/, accessed on 23 April 2023) with a confidence threshold of 70%.

### 2.3. pH, Total Sugar and Alcohol Analysis

pH values were assessed using an electronic pH meter (Jenco, Shanghai, China). The content of total sugar was quantified using a method described in the literature with a few adjustments [16]. By using a modified approach, alcohol was measured using a Gas Chromatograph (GC-2010 Plus, Shimadzu Corporation, Kyoto, Japan), and 4-methyl-2-pentanol was used as the internal standard substance [17].

### 2.4. Total Phenol, Total Flavone, and Organic Acid Analysis

The total phenol content was determined by the spectrophotometric Folin–Ciocalteu method [18]. The total flavone content was determined using an aluminum chloride colorimetric assay according to the method described by Fan [19]. By using a modified approach [20], the filtered samples were analyzed using high performance liquid chromatography systems, utilizing an injection volume of 10 µL for the filtrate. The quantification of acetic acid and glucuronic acid was achieved using a UV detector (210 nm) and a C-18 column (5 µm, 4.6 mm × 250 mm; Hitachi High-Tech Fielding Corporation, Tokyo, Japan).

### 2.5. HS-SPME-GC–MS Analysis of Volatile Compounds

The process used for the extraction of volatile chemical components from the kombucha broth was close to that reported by Wang et al. [21]. A total of 5.0 g of sample was mixed with 500 μL 1,2-dichlorobenzene (1.3048 µg/µL) as an internal standard. The mixture was shaken for 2 min, and the mixed solution was heated with a water bath at 50 °C for 10 min. Then, the fiber holder plunger was depressed, allowing the insertion of 1 cm of the coated fused-silica fiber into the head-space sample for a duration of 25 min. Then, the fiber was withdrawn out of the sample and inserted into the injection port of a gas chromatographic mass spectrometer (Agilent Technologies Inc. Palo Alto, Santa Clara, CA, USA) for the analysis of volatile components.

The GC-MS analytical procedure was based on a previous study [22]. Samples were not injected in the split mode, and helium was employed as the carrier gas (99.99%, 1.5 mL/min). In the MS system, both the ion source temperature and inlet temperature were maintained at 230 °C, and the scanned area was 35–500 *m*/*z*. The GC separation was achieved on a DB-WAX column (30 m × 0.25 mm, 0.25 µm particle size). The inlet temperature was set at 230 °C. The oven temperature program was started at an initial temperature of 40 °C, held for 3.0 min, and then increased to 75 °C at 5 °C/min, where it was maintained for 5.0 min, Subsequently, the temperature was raised at 5 °C/min to 110 °C, where it was held for 5.0 min, and finally, it was increased at 10 °C/min to 230 °C, where it was maintained for 5.0 min.

The identification of unknown volatile compounds was based on a comparison with Agilent Mass Hunter Qualitative Analysis software B.07.00 (Agilent Technologies Inc., Palo Alto, CA, USA). Volatile compounds were tentatively identified by comparing their mass spectra (MS) and the practical retention indices (RI, determined by n−Alkanes C7−C40) with information from the National Institute of Standards and Technology (NIST) library. RI was calculated with the retention time of each compound and n−Alkanes C7−C40 according to a previous study [23]. The quality of each compound was determined using the internal standard method [24], and the OAV of a compound was calculated by dividing the calculated concentrations with the sensory thresholds, which were obtained from the literature.

### 2.6. Sensory Analysis

The experimental procedures were in accordance with the Declaration of Helsinki, and were approved by the Institutional Review Board of Shaanxi Normal University (protocol code 202321019). The participants were recruited among graduate students in Shaanxi Normal University, based on their interest and availability. The graduate students in this study participated on a voluntary basis and received no compensation.

The sensory properties of the kombucha samples were determined using a 9-point scale method according to a descriptive analysis technique [25]. Each kombucha sample from a different time (Day 0, 3, 7, 10, and 14) was subsequently coded with numbers and evaluated in triplicate for overall acceptability, appearance, color, odor, and taste. Scores ranged from 1 to 9, as follows: (1) extreme dislike, (2) great dislike, (3) moderate dislike, (4) slight dislike, (5) neither liking nor dislike, (6) slight liking, (7) moderate liking, (8) great liking, and (9) extreme liking. The kombucha samples were evaluated by a selected and semi-trained sensory panel of 10 testers.

### 2.7. Statistical Analysis

All data were presented as mean ± standard deviation (SD) of triplicates. The physicochemical dates were performed using the Office 2019, and were statistically processed using SPSS software 22.0 (Version: IBM SPSS Statistics 25) (*p*-value < 0.05).

A principal coordinate analysis (PCoA) was performed on the Majorbio platform, whereas an orthogonal partial least squares discriminant analysis (OPLS-DA) and a hierarchical cluster analysis (HCA) were performed in SIMCA 14.1 [26].

A correlation network analysis was used to study the correlation between the dominant genera and flavor compounds on the Cytoscape software 3.10.0-SNAPSHOT. A significant correlation was defined as a Spearman correlation coefficient R > 0.6 and *p* values ∣*p*∣ < 0.05.

## 3. Results and Discussion

### 3.1. Dynamic Shift of Microbial Communities

Microorganisms are of significant importance in the process of kombucha fermentation. The dynamic changes in microbial community structure at different time points (Day 0, 3, 7, 10, and 14) were investigated during the fermentation of kombucha made from FBT. The effective sequences of the bacteria and fungi were 601,957 and 901,964, respectively. The bases of these effective sequences were 243,604,791 bases and 206,626,067 bases, and the average lengths of the sequences were 404 bp and 229 bp, respectively. The USEARCH11-uparse algorithm was used to divide OTU. The similarity of the OTU sequence was 0.97 and the classification confidence was 0.7. The dilution curve shows a gentle trend (Figure S1), indicating that the sequencing depth was sufficient and almost all samples were detected. The sequencing results could truly reflect the diversity of bacterial and fungal communities in kombucha made from FBT.

Microbial communities can be evaluated for richness and diversity using alpha diversity, as shown in Table 1. The Chao index is an index that reflects the richness of the microbial community, and the value is proportional to the richness of the community. The Shannon index is an index reflecting microbial community diversity, and the value is inversely proportional to community diversity [27]. During fermentation, the Chao index of the bacterial community decreased and the Shannon index decreased, indicating that the richness of the bacterial community in FBT kombucha decreased whereas the diversity increased. In contrast to the change in bacterial community, the Chao index and the Shannon index of the fungal community increased over time, indicating that the fermentation process resulted in an increase in the richness and a decrease in the diversity of the fungal community. In the later stages of fermentation, since the pH value of the kombucha broth dropped below 3.0, and the dissolved oxygen gradually decreased in the samples, fungal growth was no longer possible, and this caused a decrease in fungal diversity.

**Figure 1:**
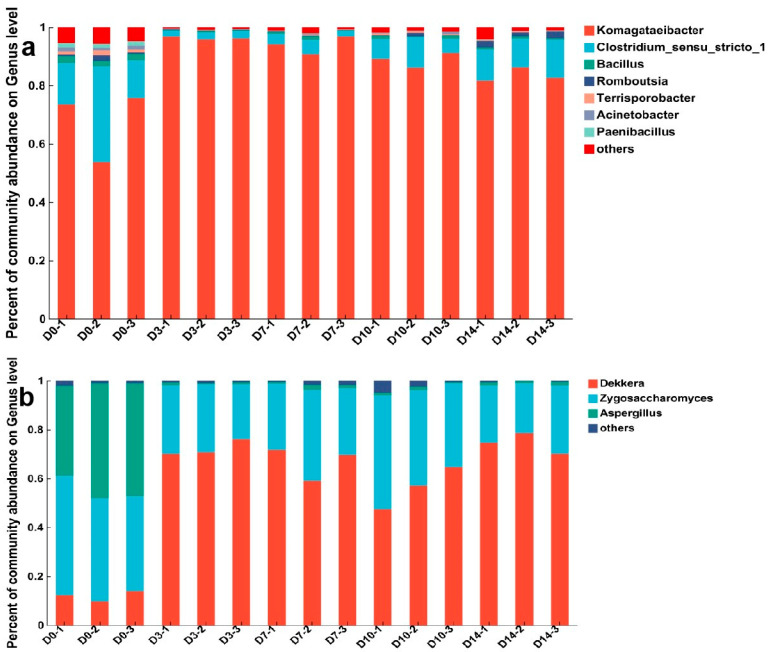
Composition of dominant microbial genera communities in kombucha made from FBT during fermentation. (**a**) The dominant bacterial genera. (**b**) The dominant fungal genera.

For microbial diversity, 24 phyla, 57 classes, 219 families, 358 genera, 111 species, and 449 OTUs in the bacterial community were identified during the kombucha fermentation process of FBT. At the genus level, several bacteria, including *Komagataeibacter*, *Clostridium sensu stricto I;*, *Bacillus*, and *Romboutsiaet*, were identified (Figure 1a). *Komagataeibacter* is responsible for the formation of cellulose biofilms in kombucha. It can also produce organic acids, such as acetic acid, glucuronic acid, gluconic acid, ascorbic acid, and some antioxidant compounds, which play an important role in contributing the sour flavor characteristics and natural antioxidant of kombucha [9]. *Komagataeibacter* dominated the whole fermentation process of kombucha, accounting for an average of 67.66–96.27% of the total bacterial population. Similar results were also obtained in a recent study, which showed that the main acetic acid bacterium found in several commercial kombucha samples was *Komagataeibacter* spp. (76.69%) [28]. During the fermentation process, the proportion of *Komagataeibacter* initially increased, reaching its peak on Day 3, followed by a slight decline. Previous research demonstrated that as the pH decreased, the survival rate of *Komagataeibacter* exhibited a significant decrease; however, *Komagataeibacter* could slow down this trend by regulating the metabolism of cell membrane fatty acids and improving its tolerance in an acidic environment [29]. Therefore, we supposed that the modest reduction in the quantity of *Komagataeibacter* after 3 days of fermentation is related to its intrinsic acid resistance, despite the pH decreasing.

In terms of the fungal community, the sequences belonged to 7 phyla, 19 classes, 64 families, 89 genera, 245 species, and 449 OTUs. At the start point, the dominant fungal genera were *Zygosaccharomyces* and *Aspergillus*, with the average relative abundances of 43.23% and 43.48%, respectively (Figure 1b). After 3 days of fermentation, the dominant fungal genera shifted to *Derkella* and *Zygosaccharomyces*, accounting for 56.43–74.39% and 24.00–30.57% of the total fungal population, respectively. *Aspergillus* is the core genus in FBT which may give FBT its special flavor and health benefits. The growth pH of *Aspergillus* was 3–6, and the most suitable growth pH is 5.0 [30]; it is therefore sensitive to low pH. During kombucha fermentation, the pH value of the broth dropped sharply, so *Aspergillus* decreased and almost stopped growing. *Zygosaccharomyces* and *Dekkera* are known to be tolerant to weak acidity and low pH [31]. *Dekkera* has the capacity to generate elevated ethanol and is associated with the production of organic acids and esters, which have a pivotal role in the formation of flavor substances in kombucha. *Dekkera* does not require high oxygen and can grow under hypoxia or low-oxygen conditions, while *Zygosaccharomyces* requires a certain oxygen supply [8]. Adequate levels of dissolved oxygen were also present during the initial day of fermentation, but since the biofilm formation on the surface and microbial growth could reduce the dissolved oxygen in the kombucha broth, it showed a slight decreased abundance while *Dekkera* exhibited a significantly increased abundance after 3 days of fermentation. In general, pH and dissolved oxygen had a certain impact on the fungal community succession in kombucha.

**Figure 2:**
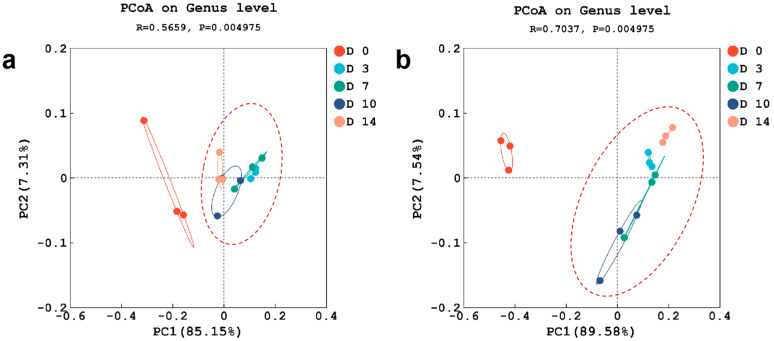
PCoA of samples of microbial genera during the fermentation of kombucha made from FBT. (**a**) The bacterial genera. (**b**) The fungal genera.

PCoA was performed to compare the difference in the microbial community during the kombucha fermentation from different time points (Figure 2). The two principal coordinate axes explained 92.46% of bacterial total variation and 97.12% of fungal total variation (Figure 2a,b). Regarding both bacterial and fungal communities, the samples collected on the 0th day formed a distinct cluster, while those from the 3rd, 7th, 10th, and 14th days were grouped separately. Zhang et al., demonstrated that some factors could influence the abundances of microbiota throughout the fermentation process [32]. Therefore, the PCoA results in this study indicated that the environmental factors after 3 days were quite different from the start point. Combined with the results and discussion of community composition, the possible factors affecting FBT kombucha could be pH and dissolved oxygen. Future studies about the possible factors affecting kombucha fermentation will enhance our understanding of the mechanisms governing microbial dynamic changes and offer possible strategies for controlling microbial abundance.

### 3.2. Dynamic Changes in the Physicochemical Properties

The dynamic changes in pH, reducing sugar, and the major chemical compounds were analyzed during the kombucha fermentation of primary FBT. As shown in Table 2, the initial pH before fermentation was about 5.16, which decreased significantly to 3.01 after 3 days of fermentation. The most suitable growth pH for *Aspergillus* is around 5.0 [30]; therefore, the abundance of *Aspergillus* was significantly decreased (Figure 1). After that, the decrease in pH was not so prominent, and it stabilized at 2.55 on Day 14, which is consistent with the research results of Chakravorty et al. [7].

The content of reducing sugar and ethanol initially increased and then decreased, reaching maximum values, which were 38.40 ± 1.16 g/L and 1.23 ± 0.03%, respectively, on Day 7. The decrease in ethanol concentration was due to its utilization by acetic acid bacteria to produce acetic acid. Acetic acid is the most abundant and most important organic acid in kombucha. Its content increased continuously during fermentation, reaching a peak of 10.57 ± 0.06 g/L at 14 days of fermentation. Glucuronic acid is one of the most important components with physiological functions in kombucha [33]. Its content initially increased and then decreased during the fermentation process, and reached a maximum value of 0.99 ± 0.05 g/L on the 7th day, which is consistent with the results of Li et al. [34].

The total phenol content increased with the extension of fermentation time; it reached 751.11 ± 41.57 mg/L on the 14th day, which may be due to the increase in acidic substances in the fermentation process, and degraded the macromolecular phenolic substances into small molecules [9]. The flavonoid content of the samples from the 0th day initially increased and then decreased, reaching a maximum value of 187.78 ± 2.95 mg/L on the 10th day.

### 3.3. Dynamic Changes in Volatile Substances

Given the significance of flavor quality in kombucha, there is a need for a more comprehensive exploration of the flavor chemical dynamics in FBT kombucha. The volatile components in the kombucha broth during fermentation were extracted using HS-SPME and subsequently analyzed using GC-MS. A total of 49 compounds were identified, and these volatile compounds were divided into six classes, including 12 alcohols, 7 acids, 16 esters, 5 aldehydes, 4 ketones, and 5 phenols (Table 3). The contents of 4-methyl-2-pentanol, heptaethylene glycol, alpha-terpineol, acetic acid, decanoic acid, ethyl acetate, phenethyl acetate, and other substances increased significantly during fermentation, indicating that these substances may be metabolites during fermentation. These volatile flavor constituents, both in terms of their composition and quantity, exhibited dynamic fluctuations throughout the fermentation process (Figure S2). Based on the pertinent literature reports, the essential components responsible for the “fungal aroma” of FBT formation are methyl salicylate (herbaceous scent), linalool (woody and fruity), and β-violone (violet and wood) [35]. These compounds were identified in our study, confirming that the introduction of FBT enhanced the flavor profile of kombucha. Some volatile flavor components increased while others decreased, thereby influencing the dynamic changes in the flavor characteristics of the kombucha fermentation broth over the entire fermentation period [4].

**Figure 3:**
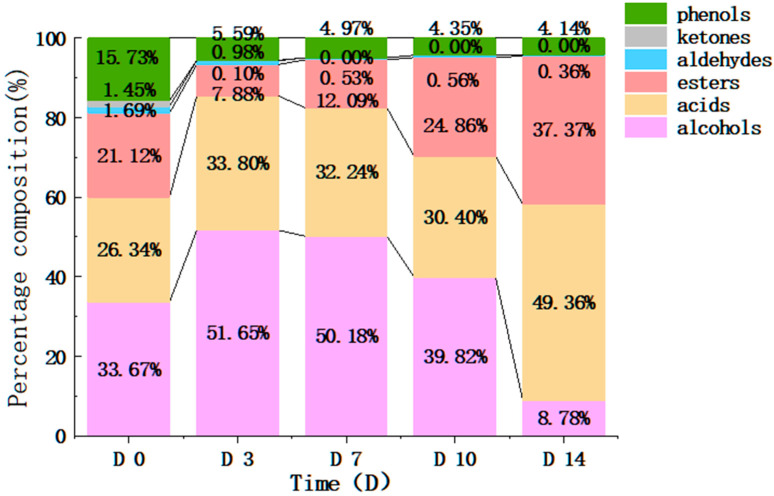
Changes in volatile percentage content in kombucha made from FBT at different times.

Before fermentation, 31 volatile flavor components were identified in kombucha, belonging to alcohols, acids, esters, aldehydes, ketones, and phenols (Table 3). The maximum content was that of alcohols (including 12 different types of alcohols), accounting for up to 33.67% of all volatile components (Figure 3). The second highest was acids, of which there were seven different types sharing 26.34% of all volatile components (Figure 3). On the 3rd day of kombucha fermentation, 29 volatile flavor components were identified (Table 3). The quantity of acid compounds and the concentration of acid and alcohol compounds exhibited substantial increases in comparison to the samples from Day 0. Among these compounds, alcohols were the most prominent, constituting as much as 51.65% of all volatile constituents, followed by acids, making up 33.80% of all volatile components (Figure 3). The increase in alcohol and acids could be due to the yeast in kombucha SCOBY converting the reducing sugar into alcohol, and then acetic acid bacteria transitioning alcohol to acids. Previous studies have shown that FBT will produce (3E,5E)-3,5-octadien-2-one, beta-ionone, trans-2,4-heptadienal, methylheptenone, and other substances during the flowering process [32]. It is therefore speculated that 13 substances, only present in the early fermentation stages (Day 0 and Day 3), such as D-alaninol, diisobutylphthalate, (3E,5E)-3,5-octadien-2-one, and methylheptenone, were from the substrate FBT.

Overall, 26, 26, and 24 volatile flavor components were identified in kombucha on the 7th, 10th, and 14th days of fermentation, respectively. During this fermentation period, there was a continuous increase in the concentration of acid and ester compounds (Figure 3). On the 7th day of kombucha fermentation, the concentration of alcohols was the highest, accounting for up to 50.18% of all volatile components. On the 14th day, the concentrations of acids and esters were the highest, accounting for up to 49.63% and 37.37% of all volatile components, respectively. It was speculated that in the mid-to-late fermentation stages, alcohols and acids form esters under the action of microorganisms [36].

The research results of Wang et al. revealed that there was a gradual reduction in aromatic components, and some even disappeared with the increase of microbial community throughout the fermentation time [20]. In this study, the diversity of bacteria increased, while the diversity of fungi decreased (Table 1). Meanwhile, the types of volatile compounds decreased, whereas the total amount initially increased and then decreased (Table 3 and Figure S3), indicating that fungi play a more important role in the change in flavor compounds in the fermentation of kombucha made from FBT.

**Figure 4:**
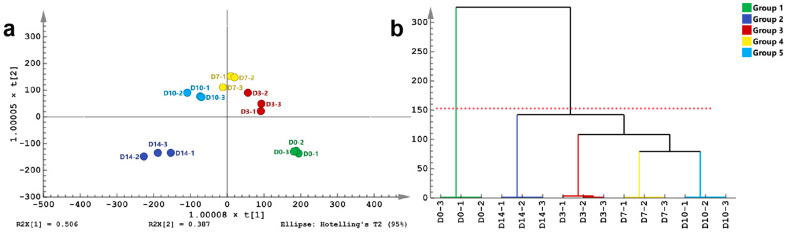
The OPLS-DA and HCA during the fermentation of kombucha made from FBT. (**a**) OPLS-DA. (**b**) HCA.

The OPLS-DA was used for the detection of volatile differences in the kombucha samples from the different fermentation time points. The Hotelling’s T2 (95%) outlier detection results showed that the samples had no strong outlier, and the cumulative variance contribution rate of the two principal components (X1 = 50.6%, X2 = 38.7%) reached 89.3%. As shown in Figure 4a, the samples from the 0th day were clustered in the right bottom quadrant, and the samples from the 3rd day, 7th day, and 10th day were clustered closely in the upper quadrant, whereas the samples from the 14th day were clustered in the left bottom quadrant, which indicated that the composition and content of volatile substances were different among the kombucha samples with different fermentation times. A similar result was also demonstrated in Pu-erh tea kombucha fermentation using principal component analysis [37]. HCA was also used to analyze the volatile substances (Figure 4b). The fermentation process was obviously divided into two groups when the Euclidean distance is 150. In the cluster diagram of volatile compounds, the samples from the 7th and 10th days belonged to one group at the minimum distance, indicating that they had the greatest similarity. The PCoA results of microbial communities were consistent with the OPLS-DA and HCA results of volatile substances, indicating that the succession of microbial communities had an important impact on the formation and changes in volatile substances.

### 3.4. Correlation of Microorganisms with Volatile Compounds

**Figure 5:**
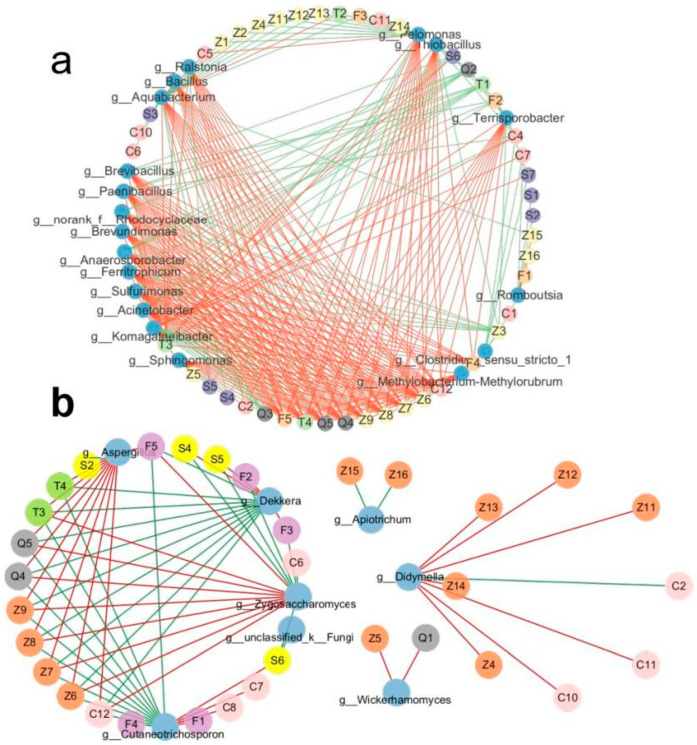
Correlation analysis between microbial genera and volatile compounds during the fermentation of kombucha made from FBT. (**a**) The bacterial genera. (**b**) The fungal genera. The red lines indicate a positive correlation, and the green lines indicate a negative correlation.

The variation in volatile compounds throughout fermentation might be associated with the changes in the microbial community of kombucha samples prepared from FBT. To investigate this connection, network correlation analysis was used to reveal the relationship between the dominant microbial genera and different volatile compounds. Regarding the bacterial genera, the predominate genus *Komagataeibacter* was positively associated with eight significantly different volatile compounds, mainly acids and alcohols (Figure 5a), which might be due to its ability to transform ethanol into acetic acid [38]. It was also reported that *Komagataeibacter* in wine was significantly and positively correlated with straight-chain fatty alcohols [39]. Meanwhile, *Komagataeibacter* was positively related to the key aroma compounds (OAV ≥ 1) (Table S1), such as linalool with floral, 3-methyl-1-butanol with floral, and octanal with green, which indicated that *Komagataeibacter* was related to the formation of alcoholic flower aroma substances. On the other hand, *Komagataeibacter* was negatively associated with 11 significantly different volatile compounds (mainly esters). A previous study showed that *Komagataeibacter* exhibited a positive relationship with esters in kombucha made from raw Pu-erh tea [12]. However, the ester content in FBT without “Golden flower” was significantly lower than that in FBT [40]. We speculated that the decrease in the population of *Aspergillus* may reduce the content of esters during fermentation and therefore affect the relationship between *Komagataeibacter* and esters in our study.

The correlation analysis between flavor substances and fungi is shown in Figure 5b. *Aspergillus* was positively associated with 10 significantly different volatile compounds, including octanal, (E, E)-2,4-heptadienal, methyl salicylate, and so on, which would provide a fatty, floral odor in FBT [41]. In this study, these compounds disappeared on Day 3, with a sharp decline in the abundance of *Aspergillus*. A previous study also reported that the “fungal flower” attributes were significantly positively related to methyl salicylate, and the increase in this aroma was possibly related to the metabolism of *Aspergillus* [6]. *Zygosaccharomyces* was positively associated with most of the same volatile substances as *Aspergillus*, and negatively associated with six significantly different volatile compounds (2-methylpropionic acid with cheese, isovaleric acid with cheese, 4-ethylphenol with spice, 4-ethylguaiacol with clove, et al.). 2-methylpropionic acid and isovaleric acid had an unpleasant burnt and cheesy smell that adversely affected the quality of kombucha. The results also showed that *Zygosaccharomyces* could prohibit cheese compound synthesis. The appropriate strengthening of the inhibitory microorganisms on cheese compound synthesis may help to control compounds in kombucha. *Dekkera* produces high levels of ethanol and is associated with the production of organic acids and esters [9]. In this study, *Dekkera* was positively associated with 2-methylpropionic acid, isovaleric acid, and phenols (4-ethylguaiacol and 4-ethylphenol). These two phenol aromas that are commonly produced by *Dekkera* yeasts are described as “medical”, “horsy”, or “smoky”, and have a negative influence on the sensorial characteristics of wine [42]. As we can see from Table 3, these two phenol compounds significantly increased on Day 3 and showed a gentle increase after that. Therefore, the fermentation could be stopped earlier in order to reduce these flavors.

### 3.5. Sensory Analysis

**Figure 6:**
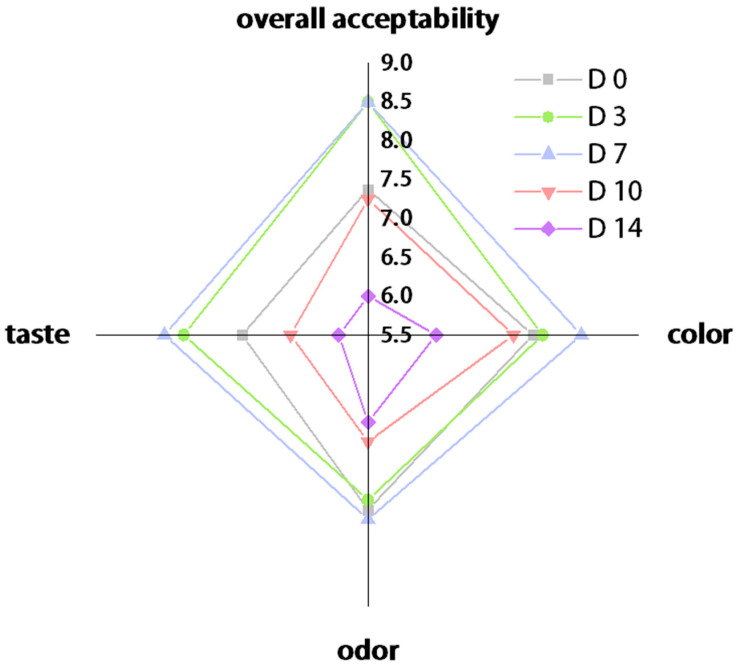
Sensory evaluation of kombucha made from FBT samples during storage fermentation.

**Figure 7:**
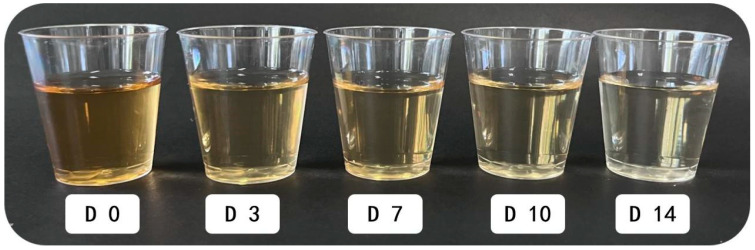
The appearance of kombucha made from FBT.

The sensory characterization of the kombucha samples was performed during the fermentation period (Figure 6). The kombucha sample from the 7th day was the most preferred beverage in terms of all sensory characteristics, and the contents of ethanol and glucuronic acid were the highest (Table 2), which provided higher functional properties. It can be observed that the color of the sample was a yellow hue and became lighter and clearer as the fermentation time increased (Figure 7). This phenomenon may be caused by microbial activity degrading certain substances [43]. According to previous studies, it has been observed that the color of Kombucha tea prepared with black tea changes from reddish brown to light brown during fermentation, which is attributed to the conversion of polyphenols, primarily theaflavin and thearubigin [7]. The main polyphenols found in FBT are theaflavins, thearubin, and theafuscin [44]. Therefore, it can be speculated that the color change in FBT kombucha is also caused by the alteration of these polyphenols.

In terms of odor, the sample from the 0th day smelled like a ‘fungal flower’, and the samples from the mid-stage (Day 3 and Day 7) had a distinctive fruity fragrance, whereas the odor of the late-stage samples (Day 10 and Day 14) changed into a sharp acetic acid. The fruity flavor in the mid-stage may be produced by acetic acid bacteria since in brewed tea leaves, they can produce an acid with a citrus flavor [45]. Overall, the FBT kombucha was deemed harmonious and enjoyable [46], with a mild vinegar-like taste and a pleasantly fruity, sour flavor in our study.

## 4. Conclusions

In this study, FBT was using for kombucha preparation for the first time. *Komagataeibacter* was the most predominant bacterium. For the dominant fungi, there was a significant shift from *Aspergillus* and *Zygosaccharomyces* to *Zygosaccharomyces* and *Derkella* after 3 days of fermentation. The physicochemical properties changed over time, and some beneficial compounds such as organic acids and polyphenols increased significantly after fermentation. Moreover, the key volatile compounds were alcohols, acids, esters, aldehydes, ketones, and phenols. There were great differences in volatile profiles among the kombucha samples with different fermentation times. Furthermore, *Komagataeibacter* displayed a positively correlation with acids and alcohols, while *Zygosaccharomyces* and *Aspergillus* from FBT were positively associated with some esters and aldehydes. This study showed that FBT was a suitable substrate for kombucha fermentation, and it had healthy biological properties. The results may provide valuable insights for better understanding the role of the microbial community in FBT kombucha and improving the flavor quality of this novel kombucha beverage.

## Figures and Tables

**Table 1:** α-diversity indexes of bacterial and fungal communities in kombucha made from FBT.

Samples	Bacteria		Fungi	
	Chao Index	Shannon Index	Chao Index	Shannon Index
D 0	190.55	1.74	28.00	1.29
D 3	181.43	1.37	29.25	1.45
D 7	161.11	0.97	34.00	1.57
D 10	153.00	0.53	35.51	1.68
D 14	151.30	0.22	37.25	1.76

**Table 2:** Changes in physicochemical properties during the fermentation process of kombucha made from FBT.

Time (d)	pH	Reducing Sugar (g/L)	Alcohol (%)	Acetic Acid (g/L)	Glucuronic Acid (g/L)	Total Phenols (mg/L)	Total Flavones (mg/L)
D 0	5.16 ± 0.01 ^a^	0.02 ± 0.00 ^c^	0.18 ± 0.00 ^d^	0.35 ± 0.02 ^e^	0.50 ± 0.01 ^d^	573.71 ± 0.42 ^b^	169.79 ± 1.00 ^cd^
D 3	3.01 ± 0.03 ^b^	10.99 ± 0.46 ^b^	1.14 ± 0.08 ^a^	1.01 ± 0.04 ^d^	0.69 ± 0.07 ^bc^	638.77 ± 17.72 ^b^	176.17 ± 2.49 ^bc^
D 7	2.69 ± 0.03 ^c^	38.40 ± 1.16 ^a^	1.23 ± 0.03 ^a^	2.81 ± 0.01 ^c^	0.99 ± 0.05 ^a^	705.43 ± 22.29 ^a^	182.65 ± 0.57 ^ab^
D 10	2.58 ± 0.01 ^d^	38.00 ± 1.57 ^a^	0.84 ± 0.02 ^b^	6.72 ± 0.01 ^b^	0.74 ± 0.05 ^b^	741.23 ± 17.20 ^a^	187.78 ± 2.95 ^a^
D 14	2.55 ± 0.02 ^d^	36.61 ± 1.55 ^a^	0.59 ± 0.00 ^c^	10.57 ± 0.06 ^a^	0.58 ± 0.00 ^cd^	751.11 ± 41.57 ^a^	161.16 ± 5.89 ^d^

The lowercase letters in the upper right corner of the data are used for significance difference analysis.

**Table 3:** Volatile compounds of the kombucha made from FBT based on gas chromatography–mass spectrometry analysis (ng/g).

Number	Volatile Compounds	RI	D 0	D 3	D 7	D 10	D 14
C1	Ethanol	952	1234.30 ± 251.89	16,301.58 ± 749.91	24,121.57 ± 1738.78	21,361.17 ± 233.76	—
C2	2-Hexadecanol	1063	288.90 ± 61.43	55.36 ± 1.44	173.15 ± 12.61	—	—
C3	4-Methyl-2-pentanol	1204	357.57 ± 34.32	802.42 ± 89.52	—	—	—
C4	3-Methyl-1-butanol	1253	2099.66 ± 335.53	5633.09 ± 461.33	5096.93 ± 134.36	3626.46 ± 247.04	2013.89 ± 106.04
C5	Hexyl alcohol	1437	63.72 ± 5.37	46.22 ± 11.83	—	—	—
C6	2-Ethylhexanol	1572	38.24 ± 9.81	214.84 ± 9.39	267.80 ± 19.37	215.44 ± 18.61	259.61 ± 12.09
C7	Linalool	1614	111.35 ± 19.82	366.89 ± 61.94	524.98 ± 27.88	409.19 ± 12.81	270.00 ± 13.87
C8	Phenethyl alcohol	1981	444.55 ± 95.79	1656.53 ± 323.66	2904.51 ± 304.26	2904.04 ± 131.65	2948.15 ± 618.25
C9	Heptaethylene glycol	2699	42.05 ± 7.03	49.62 ± 7.33	51.21 ± 1.28	61.36 ± 8.00	—
C10	alpha-Terpineol	1738	—	—	61.50 ± 1.06	71.10 ± 9.26	109.05 ± 7.67
C11	Isobutyl alcohol	1115	—	—	—	242.67 ± 20.17	124.47 ± 5.07
C12	D-Alaninol	588	244.98 ± 38.29	—	—	—	—
S1	D-Alanine	715	—	63.49 ± 17.17	1449.83 ± 207.70	1248.76 ± 80.45	—
S2	DL-Alanine	852	—	689.94 ± 53.69	450.01 ± 125.53	1225.57 ± 184.15	—
S3	Acetic acid	1522	3606.66 ± 358.55	14,238.89 ± 1534.35	19,109.59 ± 1774.21	20,850.24 ± 2779.53	29,033.70 ± 1363.81
S4	2-Methylpropionic acid	1632	65.92 ± 5.03	342.10 ± 83.67	261.86 ± 96.80	347.56 ± 12.44	514.09 ± 34.95
S5	Isovaleric acid	1715	181.25 ± 16.78	1542.36 ± 23.28	1268.29 ± 225.04	859.60 ± 455.22	2471.62 ± 264.49
S6	Octanoic acid	2094	—	256.71 ± 52.62	296.02 ± 34.11	—	180.97 ± 52.23
S7	Decanoic acid	2302	—	59.67 ± 7.41	400.28 ± 30.53	—	—
Z1	Ethyl acetate	910	2529.29 ± 353.63	3523.14 ± 531.95	6785.00 ± 1808.67	15,836.06 ± 430.06	21,176.62 ± 3496.57
Z2	Ethyl caprate	1687	—	85.26 ± 9.49	126.42 ± 17.48	146.20 ± 16.14	129.23 ± 21.65
Z3	Isobornyl acetate	1739	—	57.37 ± 12.12	—	—	—
Z4	Phenethyl acetate	1798	62.98 ± 2.07	165.47 ± 31.55	273.48 ± 17.59	646.79 ± 9.25	904.79 ± 33.09
Z5	Acetic acid, amyl ester	1130	191.50 ± 42.09	—	741.72 ± 49.37	—	—
Z6	9-Octadecen-12-ynoic acid methyl ester	1122	50.95 ± 5.55	—	—	—	—
Z7	Pentanoic acid, octylester	1290	83.57 ± 9.77	—	—	—	—
Z8	Methyl salicylate	1776	28.55 ± 0.67	—	—	—	—
Z9	Diisobutyl phthalate	2619	142.70 ± 30.62	—	—	—	—
Z10	(E)-2-Hexenyl benzoate	1581	—	—	71.18 ± 11.65	—	—
Z11	Isoamyl acetate	1130	—	—	—	1135.76 ± 109.74	1187.25 ± 184.94
Z12	Ethyl caproate	1256	—	—	—	125.00 ± 36.02	166.92 ± 6.03
Z13	Ethyl phenylacetate	1785	—	—	—	87.91 ± 2.05	163.22 ± 3.93
Z14	Ethyl palmitate	1883	—	—	—	61.18 ± 3.81	64.85 ± 8.24
Z15	Methyl acetate	863	—	—	—	—	524.42 ± 26.95
Z16	12,15-Octadecadiynoic acid methyl ester	1673	—	—	—	—	61.44 ± 4.17
Q1	1-Nonanal;	1460	59.17 ± 13.31	33.96 ± 3.54	180.16 ± 29.46	156.69 ± 20.38	—
Q2	Benzaldehyde	1581	—	186.60 ± 36.33	—	—	—
Q3	2,5-Dimethylbenzaldehyde	1792	117.60 ± 10.08	257.10 ± 8.77	167.92 ± 2.98	252.21 ± 26.84	234.24 ± 15.42
Q4	Octanal	1336	39.34 ± 7.25	—	—	—	—
Q5	trans,trans-2,4-Heptadienal	1562	30.92 ± 7.64	—	—	—	—
T1	2-Octanone, 1-nitro-	1123	—	463.76 ± 66.65	—	—	—
T2	Methylheptenone	1408	61.29 ± 12.90	49.33 ± 8.97	—	—	—
T3	(3E,5E-)3,5-Octadien-2-one	1586	81.61 ± 25.30	—	—	—	—
T4	beta-Ionone	2016	68.72 ± 12.28	—	—	—	—
F1	2,6-Di-tert-butyl-4-methylphenol	2000	1702.59 ± 113.89	2313.07 ± 240.37	2858.42 ± 172.66	2691.40 ± 41.43	2154.56 ± 149.84
F2	4-Ethylguaiacol	2060	—	76.37 ± 14.22	69.33 ± 2.92	61.59 ± 1.02	67.07 ± 3.34
F3	4-Ethylphenol	2160	40.72 ± 3.57	220.94 ± 38.81	243.99 ± 23.08	234.85 ± 5.91	283.27 ± 21.11
F4	2,4-Di-tert-butylphenol	2299	538.04 ± 28.05	108.16 ± 12.78	116.04 ± 25.11	167.73 ± 5.30	198.28 ± 5.34
F5	Azulene	1753	20.53 ± 0.18	—	—	—	—

## Data Availability

The data presented in this study are available on request from the corresponding author.

## Supplementary Materials

The following supporting information can be downloaded at: https://www.mdpi.com/article/10.3390/foods12234242/s1, Figure S1: Dilution curves of bacteria (a) and fungi (b) during the fermentation process of kombucha made from FBT; Figure S2: Heatmap of the dynamics of volatile substances during the fermentation of kombucha made from FBT; Figure S3: Changes of kombucha made from FBT content in different fermentation times; Table S1: OAV and aroma description of aroma substances in kombucha made from FBT.
