# Supplementary material for: Dynamic Changes in Microbial Communities, Physicochemical Properties, and Flavor of Kombucha Made from Fu-Brick Tea
Source: Foods. 2023 Nov 24;12(23):4242. doi: 10.3390/foods12234242 (PMC10706452; doi:10.3390/foods12234242)
Supplement: Supplementary file 1 [file foods-12-04242-s001.zip › foods-2681783-supplementary.pdf]

Supplementary

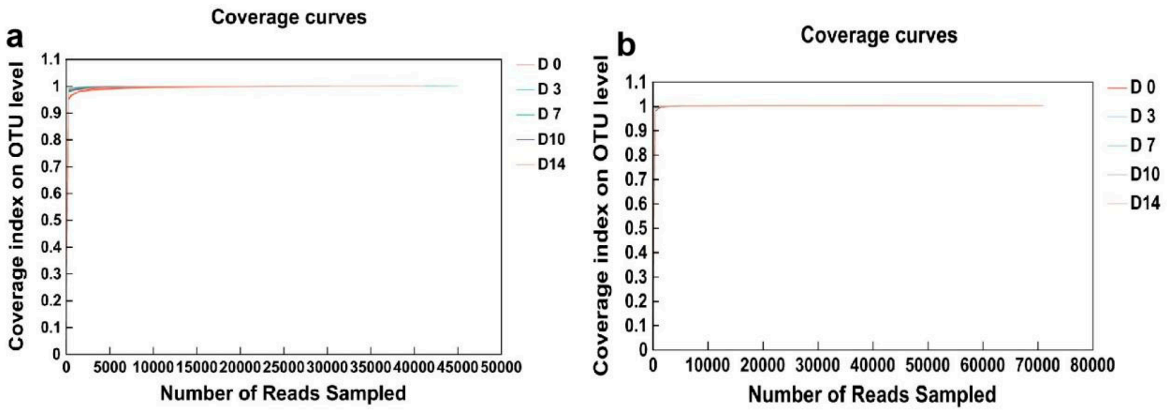

Figure S1. Dilution curves of bacteria (a) and fungi (b) during the fermentation process of kombucha made from FBT.

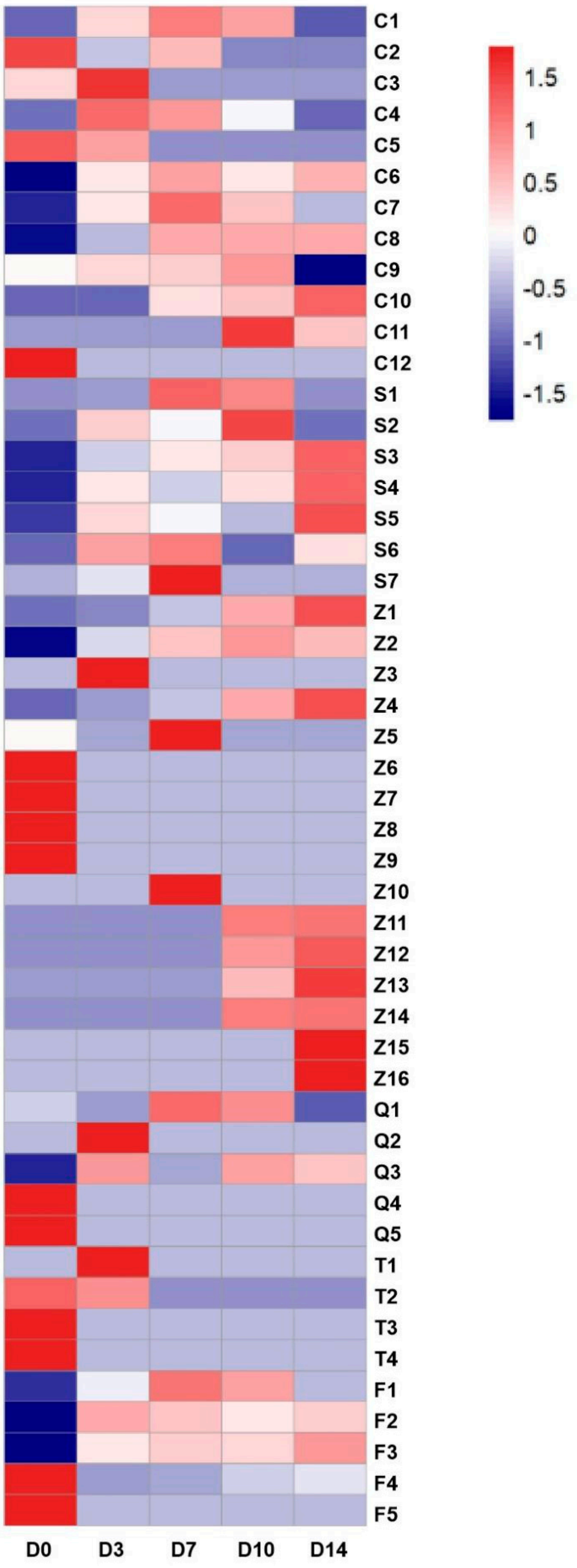

Figure S2. Heatmap of the dynamics of volatile substances during the fermentation of kombucha made from FBT.

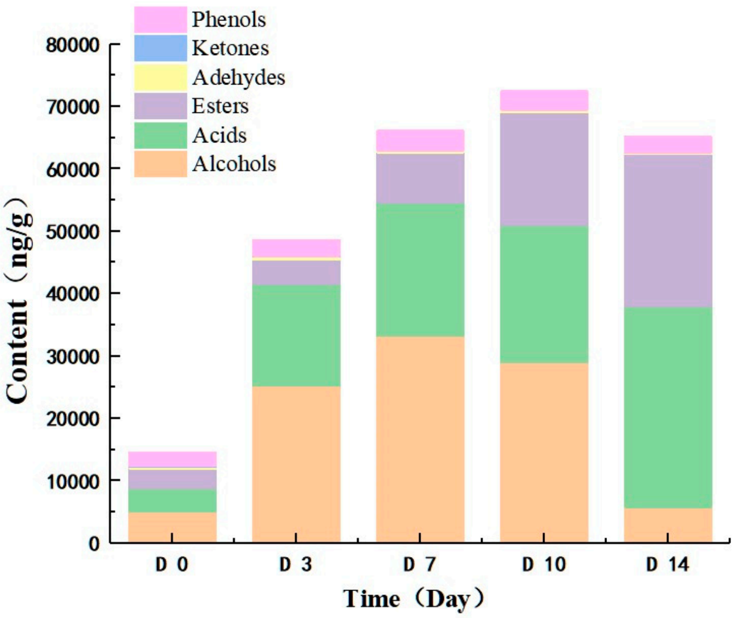

Figure S3. Changes of kombucha made from FBT content in different fermentation times.

Table S1 OAV and aroma description of aroma substances in kombucha made from FBT.

Serial number	Flavour components	Threshold value（μg/kg)	D 0	D 3	D 7	D 10	D 14	Flavor
C4	3-Methyl-1-butanol	980	2.14±0.34	5.75±0.47	5.2±0.14	3.7±0.25	2.05±0.11	Floral, Malt
C7	Linalool	25	4.45±0.79	14.68±2.48	21±1.12	16.37±0.51	10.8±0.55	Coriander, Floral, Lavender, Lemon, Rose
C8	Phenethyl alcohol	390	1.14±0.25	4.25±0.83	7.45±0.78	7.45±0.34	7.56±1.59	Fruit, Honey, Lilac, Rose, Wine
C10	alpha-Terpineol	86	—	—	<1	<1	1.27±0.09	Anise, Fresh, Mint, Oil
S3	Acetic acid	1200	3.01±0.3	11.87±1.28	15.92±1.48	17.38±2.32	24.19±1.14	Acid, Fruit, Pungent, Sour, Vinegar
S4	2-Methylpropionic acid	50	1.32±0.1	6.84±1.67	5.24±1.94	6.95±0.25	10.28±0.7	Burnt, Butter, Cheese, Sweat
S5	Isovaleric acid	490	<1	3.15±0.05	2.59±0.46	1.75±0.93	5.04±0.54	Cheese, Pungent
Z1	Ethyl acetate	7500	<1	<1	<1	2.11±0.06	2.82±0.47	Aromatic, Brandy, Grape
Z2	Ethyl caprate	20	—	4.26±0.47	6.32±0.87	7.31±0.81	6.46±1.08	Brandy, Grape, Pear
Z4	Phenethyl acetate	250	<1	<1	1.09±0.07	2.59±0.04	3.62±0.13	Flower, Honey, Rose
Z5	Acetic acid, amyl ester	43	4.45±0.98	—	17.25±1.15	—	—	Apple, Banana, Glue, Pear
Z11	Isoamyl acetate	30	—	—	—	37.86±3.66	39.57±6.16	Apple, Banana, Glue, Pear
Z12	Ethyl caproate	5	—	—	—	25±7.20	33.38±1.21	Apple Peel, Brandy, Fruit Gum, Overripe Fruit, Pineapple
Z13	Ethyl phenylacetate	155.55	—	—	—	<1	1.05±0.03	Floral, Fruit, Honey, Rose
Q1	1-Nonanal;	15	3.94±0.89	2.26±0.24	12.01±1.96	10.45±1.36	—	Floral, Green, Lemon
Q3	2,5-Dimethylbenzaldehyde	200	<1	1.29±0.04	<1	1.26±0.13	1.17±0.08	—
Q4	Octanal	6.9	5.7±1.05	—	—	—	—	Soap, lemon, green
T4	beta-Ionone	3.5	19.64±3.51	—	—	—	—	Violet, Floral,
F1	2,6-Di-tert-butyl-4-methylphen	1000	1.7±0.11	2.31±0.24	2.86±0.17	2.69±0.04	2.15±0.15	Toasted Cereal

	ol							
F2	4-Ethylguaiacol	16	—	4.77±0.89	4.33±0.18	3.85±0.06	4.19±0.21	Clove
F3	4-Ethylphenol	21	1.94±0.17	10.52±1.85	11.62±1.1	11.18±0.28	13.49±1.01	Phenol, Spice
F4	2,4-Di-tert-butylphenol	500	1.08±0.06	<1	<1	<1	<1	—
